# Tumor-associated macrophage polarization promotes the progression of esophageal carcinoma

**DOI:** 10.18632/aging.202201

**Published:** 2020-12-15

**Authors:** Xin Yuan, Ya Li, An Zhi Zhang, Chen Hao Jiang, Fan Ping Li, Yu Fang Xie, Jiang Fen Li, Wei Hua Liang, Hai Jun Zhang, Chun Xia Liu, Li Juan Pang, Xi Hua Shen, Feng Li, Jian Ming Hu

**Affiliations:** 1 Department of Pathology and Key Laboratory for Xinjiang Endemic and Ethnic Diseases (Ministry of Education), Department of Pathology, The First Affiliated Hospital, Shihezi University School of Medicine, Xinjiang 832000, China; 2 Department of Pathology, Beijing Chaoyang Hospital, Capital Medical University, Beijing 100020, China

**Keywords:** tumour-associated macrophage, FGL2, esophageal carcinoma, immunotherapy, tumour-infiltrating

## Abstract

The immune response facilitated by tumor-associated macrophages is a vital determinant of tumor progression. We identified differentially expressed genes between various macrophage phenotypes in the Gene Expression Omnibus, and used Kaplan-Meier Plotter to determine which of them altered the prognosis of esophageal carcinoma patients. Fibrinogen-like protein 2 (*FGL2*), an immunosuppressive factor in the tumor microenvironment of various cancers, was upregulated in M2 macrophages, and higher *FGL2* expression was associated with poorer survival in esophageal carcinoma patients. Using the TIMER database, we found that *FGL2* expression correlated positively with the levels of immune markers of infiltrating B cells, CD8+ T cells, CD4+ T cells, macrophages, neutrophils and dendritic cells in esophageal carcinoma samples. Correlation analyses in cBioPortal revealed that the mRNA levels of *FGL2* correlated strongly with those of interleukin 10, matrix metalloproteinase 9, C-C motif chemokine ligand 5, T-cell immunoglobulin mucin 3, interleukin 13, vascular cell adhesion molecule 1, macrophage colony-stimulating factor and fibroblast growth factor 7 in esophageal carcinoma tissues. The same cytokines were upregulated when esophageal squamous cell carcinoma cells were co-cultured with M2-like tumor-associated macrophages. Thus, by secreting FGL2, M2-like tumor-associated macrophages may create an immunosuppressive tumor microenvironment that induces the occurrence and progression of esophageal carcinoma.

## INTRODUCTION

Esophageal carcinoma (ESCA) is the sixth leading cause of cancer-related death and the eighth most common malignancy worldwide [1]. The incidence of esophageal squamous cell carcinoma (ESCC) is increasing globally. Although the diagnosis and treatment of ESCA have gradually improved, the five-year survival rate of ESCA patients is still only 10-20% [2], mainly due to late diagnosis, metastasis and immune resistance. The immune escape and immunosuppressive activities of tumor cells are important contributors to the progression of ESCA and the poor prognoses of ESCA patients. Thus, immunotherapy is considered a promising treatment strategy for ESCA [3].

Tumor growth is a gradual process, and its rate and outcomes depend on the interactions between tumor cells and surrounding cells in the complex and variable tumor microenvironment (TME). The functional state of immune cells in the TME is a key determinant of tumor reversion. Therefore, there is an urgent need to understand the immunophenotypes involved in tumor immunity and to identify novel immune-related therapeutic targets in ESCA.

Tumor-associated macrophages (TAMs) exert multiple effects on tumor cells in the TME. TAMs have a high degree of plasticity, and can be polarized by diverse stimuli into classically activated proinflammatory macrophages (M1-like TAMs) or alternatively activated immunosuppressive macrophages (M2-like TAMs). Polarized M1 and M2 macrophages reflect the two extremes of TAM function, as M2 macrophages are associated with the occurrence and progression of cancer and the poor prognosis of cancer patients [4, 5]. Indeed, we previously demonstrated that M2 macrophages in the TME promoted the growth, invasion and migration of ESCA cells, and were associated with a poor patient prognosis [6, 7]. Particular cytokines in the TME induce TAMs to obtain a tumor-promoting (M2) phenotype and thus exert immunosuppressive effects [8]. However, TAMs can be reprogrammed phenotypically to elicit an anti-tumor response (i.e., as M1 macrophages) [9].

Fibrinogen-like protein 2 (FGL2), a member of the fibrinogen superfamily, exerts prothrombin activity and multiple immunoregulatory functions. FGL2 is highly expressed in various tumor tissues, and is an important determinant of tumor occurrence and progression [10–12]. FGL2 functions multimodally in innate and acquired immune responses, as it enhances the activation of T regulatory cells (Tregs) and maintains their immunosuppressive activity, balances the ratio of T-helper 1 (Th1) and Th2 cells, inhibits antigen-presenting activity [13, 14], reduces the proliferation of effector T cells, suppresses the maturation of dendritic cells (DCs) [15, 16], promotes the activation of macrophages and induces the apoptosis of B cells [17]. Zhang et al. demonstrated that high FGL2 expression was an independent poor prognostic factor in renal carcinoma patients, while silencing *FGL2* significantly reduced renal cancer cell viability and increased cancer cell apoptosis [18]. In the TME, FGL2 can promote the transformation of M1 macrophages into tumor-promoting M2 macrophages by inducing CD39 expression [19]. However, the effects of FGL2 on the progression and tumor immunology of ESCA have yet to be reported.

In this study, we searched for differentially expressed genes (DEGs) between macrophages of different phenotypes to identify genes that might promote the progression of ESCA. Based on our findings, we analyzed the correlation of *FGL2* expression with the prognosis of ESCA patients and the levels of tumor-infiltrating immune cells (TIICs) and cytokines in the esophageal cancer microenvironment. Finally, we assessed the enriched Gene Ontology (GO) functions and Kyoto Encyclopedia of Genes and Genomes (KEGG) pathways of the DEGs between M1 and M2 macrophages. Our findings have revealed strategies for reversing the polarization of TAMs and developing immunotherapies for ESCA.

## RESULTS

### Distribution of TIICs in the ESCA microenvironment

**Figure 1:**
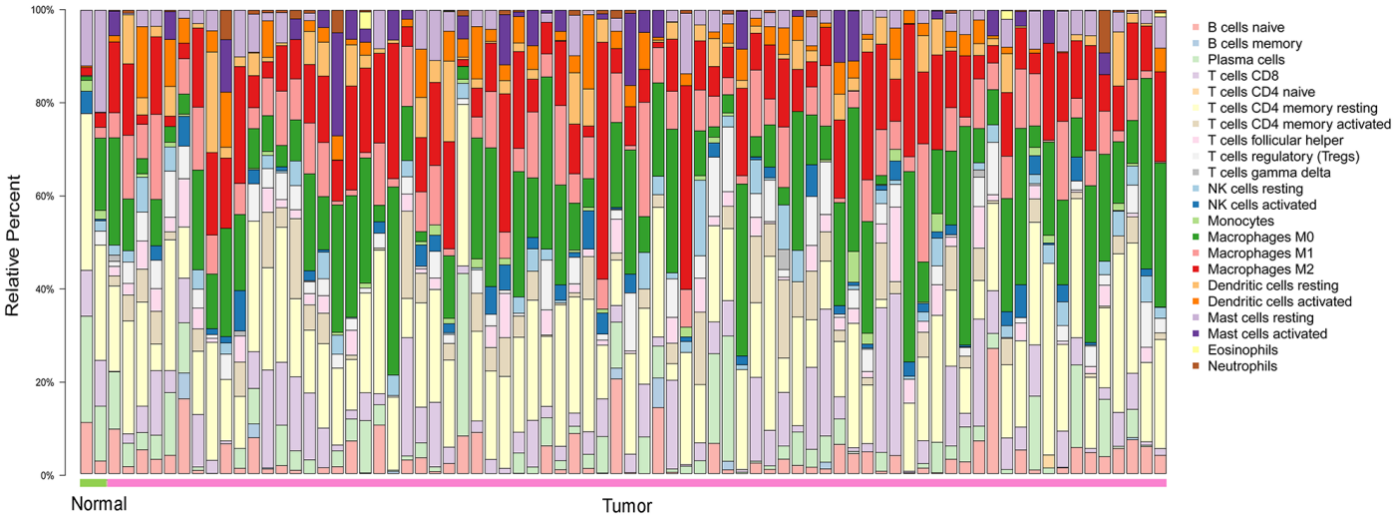
**The landscape of 22 TIICs in ESCA, and their proportions in each sample as quantified using CIBERSORT.** Normal = 2 samples; tumor = 76 samples. M0, green; M1, pink; M2, red.

To explore the distribution of TIICs in the ESCA microenvironment, we analyzed ESCA samples in The Cancer Genome Atlas (TCGA). We found that 70-80% of the TIICs in the ESCA microenvironment were macrophages and T cells with different phenotypes (Figure 1).

### Identification of DEGs between macrophages of different phenotypes

**Figure 2:**
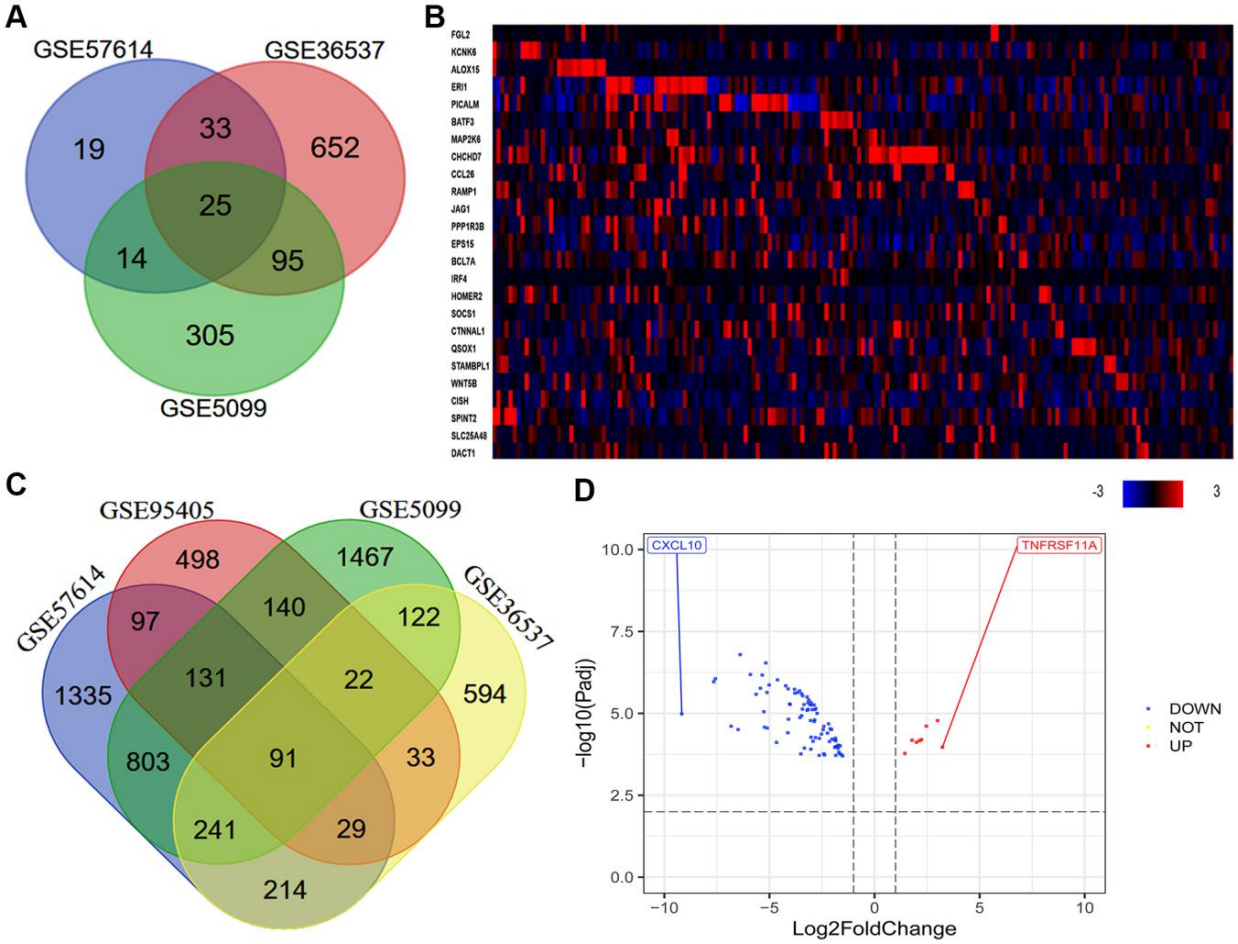
**Quantitative analysis of DEGs between macrophages of different phenotypes.** (**A**) In total, 25 consistently DEGs between M0 macrophages and M2 macrophages were identified in three datasets (GSE57614, GSE36537 and GSE5099) based on a |logFC| ≥ 1.0 and an adjusted P-value < 0.05. (**B**) In total, 91 consistently DEGs between M1 and M2 macrophages were identified in four datasets (GSE57614, GSE36537, GSE5099 and GSE95405) based on a |logFC| ≥ 1.0 and an adjusted P-value < 0.01. (**C**) The levels of the 25 consistently DEGs in ESCA samples from TCGA (tumor = 184) are shown as a heatmap, as quantified using cBioPortal. High, medium and low gene levels are represented in red, black and blue, respectively. (**D**) Volcano plot of the 91 consistently DEGs between M1 and M2 macrophages. The red spots represent the 8 upregulated genes and the blue spots indicate the 83 downregulated genes in M2 macrophages compared with M1 macrophages.

Our previous research indicated that a high density of M2 macrophages in the TME was associated with the occurrence and progression of ESCC, and with a poor prognosis in ESCC patients [6]. Thus, we used various macrophage datasets from the Gene Expression Omnibus (GEO) to identify genes that were differentially expressed between macrophages of different phenotypes. As shown in the Venn diagram in Figure 2A, we identified 25 consistently DEGs between M0 macrophages and M2 macrophages in three datasets (GSE57614, GSE36537 and GSE5099). Coincidentally, all 25 DEGs were highly expressed in samples of M2 macrophages. Next, we used cBioPortal to assess the expression of these 25 DEGs in ESCA tissues. The results revealed that some of the DEGs were highly expressed in ESCA tissues (Figure 2B). In addition, we identified 91 consistently DEGs between M1 and M2 macrophages in four datasets (GSE57614, GSE36537, GSE5099 and GSE95405) (Figure 2C). A volcano plot of the 91 DEGs demonstrated that tumor necrosis factor receptor superfamily member 11a (*TNFRSF11A*) was the most upregulated gene and C-X-C motif chemokine ligand 10 (*CXCL10*) was the most downregulated gene in M2 macrophages compared to M1 macrophages (Figure 2D).

### *FGL2* expression correlates with the level of immune cell infiltration in ESCA

**Figure 3:**
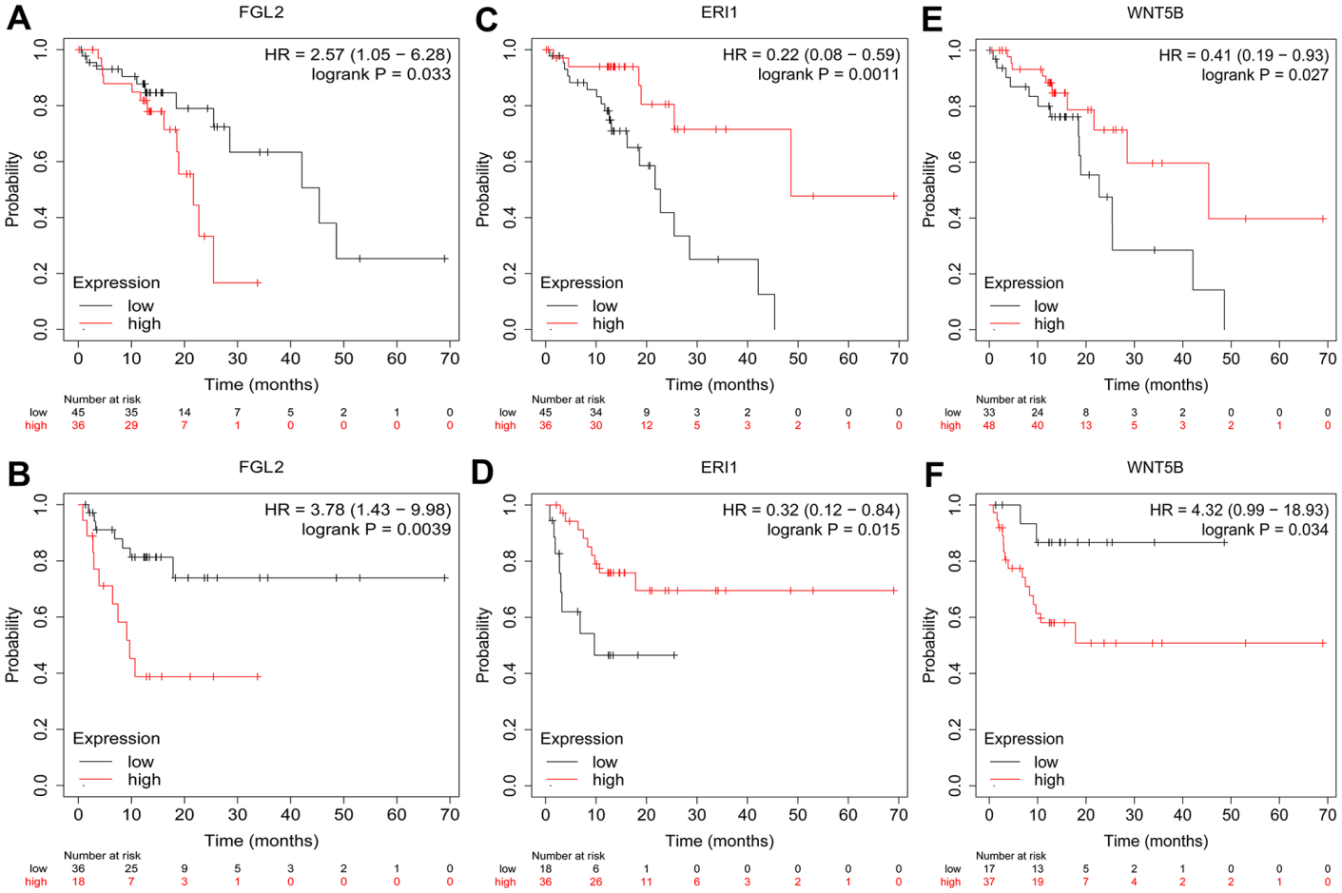
**Kaplan-Meier Plotter survival curves.** The plots display the effects of *FGL2* expression on OS (**A**), *FGL2* expression on RFS (**B**), *ERI1* expression on OS (**C**), *ERI1* expression on RFS (**D**), *WNT5B* expression on OS (**E**) and *WNT5B* expression on RFS (**F**) in ESCC patients (n = 81). P < 0.05 was considered statistically significant.

To investigate whether the DEGs between M0 macrophages and M2 macrophages were associated with the prognosis of esophageal cancer patients, we used the Kaplan-Meier Plotter online platform to assess the survival of ESCC patients based on their expression of the 25 DEGs. We found that *FGL2*, exoribonuclease 1 (*ERI1*) and *WNT5B* levels were associated with the overall survival (OS) and relapse-free survival (RFS) of ESCC patients. Specifically, higher *FGL2* expression was marginally associated with a poorer prognosis in ESCC patients (OS hazard ratio [HR] = 2.57, 95% confidence interval [CI] = 1.05 to 6.28, P = 0.033; RFS HR = 3.78, 95% CI = 1.43 to 9.98, P = 0.0039) (Figure 3A, 3B). However, lower *ERI1* expression was associated with a poorer prognosis (OS HR = 0.22, 95% CI = 0.08 to 0.59, P = 0.0011; RFS HR = 0.32, 95% CI = 0.12 to 0.84, P = 0.015) (Figure 3C, 3D). Likewise, lower *WNT5B* expression was associated with a poorer prognosis (OS HR = 0.41, 95% CI = 0.19 to 0.93, P = 0.027; RFS HR = 4.32, 95% CI = 0.99 to 18.93, P = 0.034) (Figure 3E, 3F). Information about the functions of FGL2, ERI1 and WNT5B can be found in Table 1.

**Table 1:** Functional roles of FGL2, ERI1 and WNT5B.

**Gene symbol**	**Full name**	**Function**
FGL2	Fibrinogen-like 2	This protein was cloned from cytotoxic T lymphocytes and showed 36% homology to fibrinogen β and γ chains, a member of the fibrinogen super family. It is a pleiotropic cytokine that impacts diverse cellular functions.
ERI1	Exoribonuclease 1	RNA exonuclease that binds to the 3'-end of histone mRNAs and degrades them, suggesting that it plays an essential role in histone mRNA decay after replication. Also, able to degrade the 3'-overhangs of short interfering RNAs (siRNAs) in vitro, suggesting a possible role as regulator of RNA interference (RNAi).
WNT5B	Wnt-family member 5B	This gene is a member of the WNT gene family. It encodes a protein which shows 94% and 80% amino acid identity to the mouse WNT5B protein and the human WNT5A protein, respectively. These proteins have been implicated in oncogenesis and in several developmental processes.

**Figure 4:**
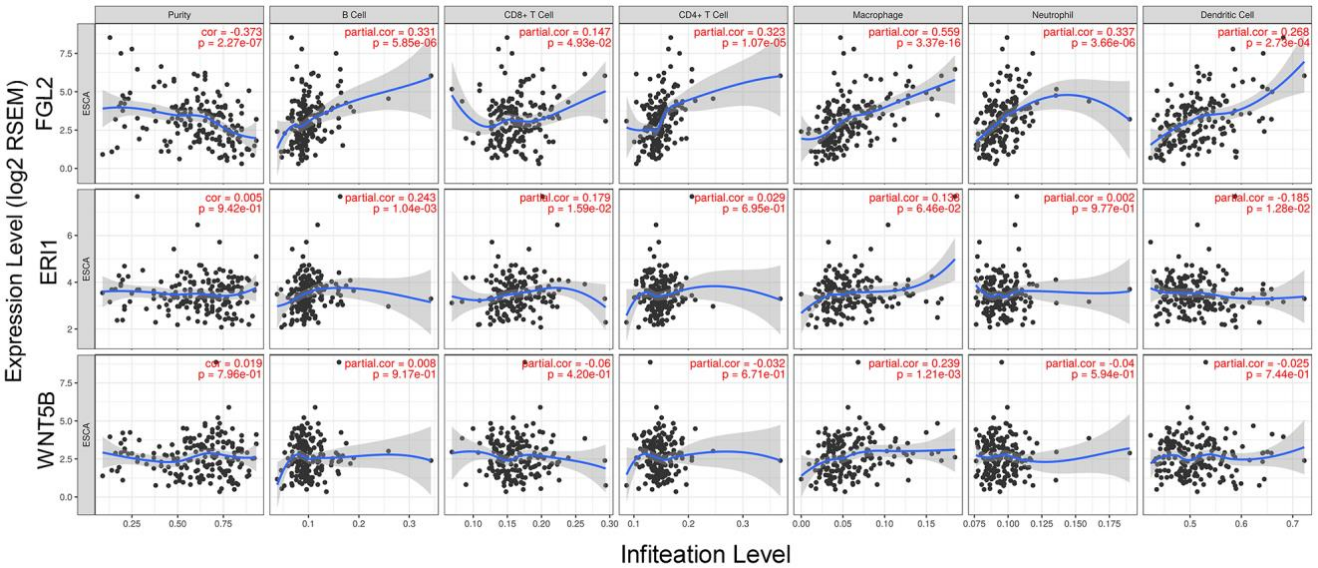
**Correlation of *FGL2*, *ERI1* and *WNT5B* levels with infiltrating immune cell levels in ESCA.** *FGL2* expression exhibited a significant negative correlation with ESCA tumor purity, and significant positive correlations with the infiltrating levels of B cells, CD8+ T cells, CD4+ T cells, macrophages, neutrophils and DCs. *ERI1* and *WNT5B* expression did not correlate significantly with ESCA tumor purity. *ERI1* expression correlated weakly with the infiltrating levels of B cells, CD8+ T cells and DCs. *WNT5B* expression only correlated weakly with macrophage levels. P < 0.05 was considered statistically significant.

Next, we used the Tumor IMmune Estimation Resource (TIMER) to assess whether *FGL2*, *ERI1* or *WNT5B* expression correlated with infiltrating immune cell levels in ESCA. Tumor purity significantly influences the genomic analysis of immune cell infiltration in clinical tumor samples [20]. As shown in Figure 4, *FGL2* expression correlated negatively with ESCA tumor purity and positively with the infiltrating levels of B cells (r = 0.331, P = 5.85e-06), CD8+ T cells (r = 0.147, P = 4.93e-02), CD4+ T cells (r = 0.323, P = 1.07e-05), macrophages (r = 0.559, P = 3.37e-16), neutrophils (r = 0.337, P = 3.66e-06) and DCs (r = 0.268, P = 2.73e-04). However, *ERI1* expression did not correlate significantly with ESCA tumor purity, and only correlated weakly with the infiltrating levels of B cells (r = 0.243, P = 10.4e-03), CD8+ T cells (r = 0.179, P = 1.59e-02) and DCs (r = -0.185, P = 1.28e-02). Similarly, *WNT5B* expression did not correlate significantly with ESCA tumor purity, and only correlated weakly with the infiltrating levels of macrophages (r = 0.239, P = 1.21e-03). These findings strongly suggested that FGL2 promotes the infiltration of immune cells, especially macrophages, in ESCA.

### Correlation analysis between *FGL2* expression and immune cell marker sets

To explore the relationship between FGL2 and various infiltrating immune cells, we used the TIMER and Gene Expression Profiling Interactive Analysis (GEPIA) databases to examine the correlations between the levels of *FGL2* and markers of various immune cells (monocytes, TAMs, M1 and M2 macrophages, B cells, T cells [general], CD8+ T cells, neutrophils, natural killer cells and DCs) and different functional T cells (Tregs, exhausted T cells, Th1, Th2, Tfh and Th17 cells) in ESCA samples (Table 2). After adjusting for purity, we found that *FGL2* levels correlated significantly with the marker levels for most immune cells and different T cells in ESCA samples (Table 2). Higher *FGL2* expression in M2 macrophages was associated with greater DC and CD8+ T cell infiltration in ESCA tissues, which was notable because DCs can promote tumor metastasis by reducing CD8+ T cell cytotoxicity and increasing Treg numbers [21]. We also found significant correlations between the levels of *FGL2* and markers of exhausted T cells and Tregs, such as programmed cell death 1 (*PD-1*), cytotoxic T-lymphocyte associated protein 4 (*CTLA4*), lymphocyte activation gene 3 (*LAG3*), T-cell immunoglobulin mucin 3 (*TIM-3*), forkhead box P3 (*FOXP3*), C-C motif chemokine receptor 8 (*CCR8*) and signal transducer and activator of transcription 5B (*STAT5B)* (Table 2). These results confirmed that *FGL2* expression correlates with infiltrating immune cell levels in ESCA.

**Table 2:** Correlation analysis between FGL2 and related immune cell genes and markers using TIMER.

**Description**	**Gene markers**	**ESCA**			
		**None**		**Purity**	
		**Cor**	**P**	**Cor**	**P**
Monocyte	CD86	0.683	***	0.637	***
	CD115 (CSF1R)	0.779	***	0.752	***
	CD14	0.553	***	0.489	***
TAM	CD68	0.328	***	0.269	**
	IL10	0.522	***	0.479	***
	CCL2	0.578	***	0.532	***
M1 Macrophage	IRF5	0.058	0.431	0.015	0.84
	INOS (NOS2)	-0.06	0.419	-0.029	0.696
	COX2 (PTGS2)	0.13	0.03	0.135	0.071
M2 Macrophage	CD163	0.66	***	0.616	***
	VSIG4	0.647	***	0.604	***
	MS4A6A	0.727	***	0.687	***
Treg	FOXP3	0.663	***	0.621	***
	CCR8	0.662	***	0.622	***
	STAT5B	0.395	***	0.431	***
	CD4	0.778	***	0.744	***
T cell exhaustion	PD-1 (PDCD1)	0.647	***	0.597	***
	CTLA4	0.646	***	0.588	***
	LAG3	0.602	***	0.554	***
	TIM-3(HAVCR2)	0.753	***	0.718	***
	GZMB	0.531	***	0.462	***
CD8+ T cell	CD8A	0.634	***	0.585	***
	CD8B	0.583	***	0.54	***
T cell (general)	CD3D	0.609	***	0.54	***
	CD3E	0.642	***	0.574	***
	CD2	0.662	***	0.606	***
B cell	CD19	0.356	***	0.248	**
	CD79A	0.427	***	0.333	***
Neutrophils	CD66b(CEACAM8)	0.028	0.707	-0.008	0.91
	CD11b (ITGAM)	0.488	***	0.421	***
	CCR7	0.515	***	0.435	***
Natural killer cell	KIR2DL1	0.271	**	0.192	*
	KIR2DL3	0.302	***	0.278	**
	KIR2DL4	0.394	***	0.342	***
	KIR3DL1	0.281	**	0.224	*
	KIR3DL2	0.22	*	0.153	0.039
	KIR3DL3	-0.04	0.59	-0.041	0.581
	KIR2DS4	0.2	*	0.183	0.014
Dendritic cell	HLA-DPB1	0.688	***	0.635	***
	HLA-DQB1	0.424	***	0.35	***
	HLA-DRA	0.604	***	0.548	***
	HLA-DPA1	0.668	***	0.623	***
	BDCA-1(CD1C)	0.576	***	0.499	***
	BDCA-4(NRP1)	0.624	***	0.595	***
	CD11c (ITGAX)	0.626	***	0.55	***
Th1	T-bet (TBX21)	0.638	***	0.568	***
	STAT4	0.672	***	0.61	***
	STAT1	0.437	***	0.399	***
	IFN-γ (IFNG)	0.519	***	0.462	***
	TNF-α (TNF)	0.165	0.025	0.106	0.155
Th2	GATA3	0.372	***	0.311	***
	STAT6	0.081	0.273	0.113	0.131
	IL13	0.324	***	0.245	**
	STAT5A	0.405	***	0.37	***
	IL2RA (CD25)	0.649	***	0.606	***
	IL2RB (CD122)	0.687	***	0.648	***
Tfh	BCL6	0.197	*	0.2	*
	IL21	0.252	**	0.188	0.012
Th17	STAT3	0.226	*	0.203	*
	IL17A	-0.009	0.907	-0.008	0.919
	IL6	0.352	***	0.307	***

Th, T helper cell; Tfh, T follicular helper cell; Treg, T regulatory cell; Cor, R value of Spearman’s correlation; None, correlation without adjustment; Purity, correlation adjusted by purity. * P < 0.01; ** P < 0.001; *** P < 0.0001.

**Figure 5:**
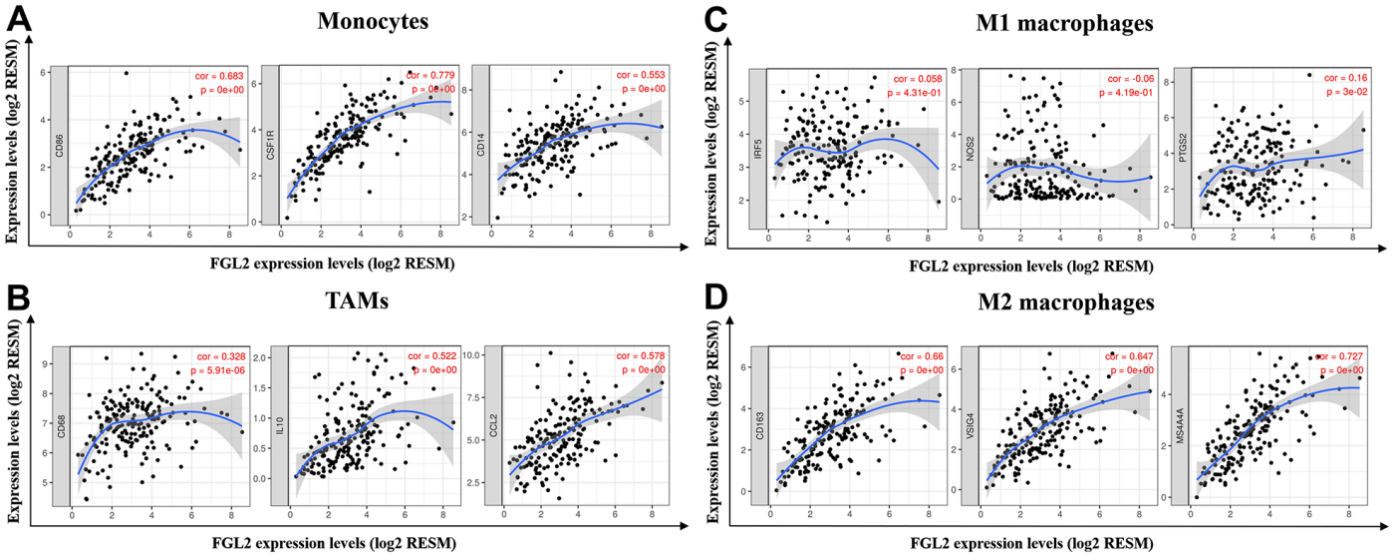
***FGL2* expression correlated with TAM polarization in ESCA.** (**A**–**D**) Scatterplots depicting the correlations between the levels of *FGL2* and genetic markers of monocytes (**A**), TAMs (**B**), M1 macrophages (**C**) and M2 macrophages (**D**). The following immune cell markers were assessed: *CD86*, *CSF1R* and *CD14* of monocytes; *CD68*, *CCL2* and *IL-10* of TAMs; *IRF5*, *NOS2* and *PTGS2* of M1 macrophages; and *CD163*, *VSIG4* and *MS4A4A* of M2 macrophages. P < 0.05 was considered statistically significant.

Next, we constructed scatterplots to depict the correlations between the levels of *FGL2* and markers of monocytes (e.g., *CD86*, *CSF1R* and *CD14*) and different macrophage phenotypes (e.g., *CD68*, *CCL2* and *IL-10* of TAMs; *IRF5*, *NOS2* and *PTGS2* of M1 macrophages; *CD163*, *VSIG4* and *MS4A4A* of M2 macrophages) in ESCA samples, as shown in Figure 5. Interestingly, the results from the TIMER database demonstrated that the levels of all the monocyte, TAM and M2 macrophage marker sets correlated strongly with *FGL2* levels (Figure 5A, 5B, 5D), while M1 macrophage marker levels did not correlate with *FGL2* levels in ESCA (Figure 5C). Similar correlations between *FGL2* expression and monocyte, TAM and M2 macrophage marker levels in ESCA samples were found using the GEPIA database (Table 3). These data verified the results from the GEO database, indicating that FGL2 may promote the polarization of TAMs into M2 macrophages in ESCA.

**Table 3:** Correlation analysis between FGL2 and markers of monocyte and different phenotypes macrophages in GEPIA.

**Description**	**Gene markers**	**ESCA**			
		**Tumor**		**Normal**	
		**R**	**P**	**R**	**P**
Monocyte	CD86	0.68	***	0.65	0.017
	CD115 (CSF1R)	0.77	***	0.58	0.043
	CD14	0.54	***	0.74	*
TAM	CD68	0.3	***	0.42	0.15
	IL10	0.53	***	0.82	**
	CCL2	0.58	***	0.82	*
M1 Macrophage	IRF5	0.029	0.7	0.33	0.27
	INOS (NOS2)	-0.066	0.38	0.42	0.16
	COX2 (PTGS2)	0.16	0.028	0.75	*
M2 Macrophage	CD163	0.63	***	0.87	**
	VSIG4	0.64	***	0.8	*
	MS4A6A	0.72	***	0.81	*

Tumour, correlation analysis in tumour tissue of TCGA; Normal, correlation analysis in normal tissue of TCGA. * P < 0.01; ** P < 0.001; *** P < 0.0001.

Together, the above findings suggested that M2 macrophages may regulate immune cell infiltration by secreting the immunosuppressive factor FGL2, thereby producing a microenvironment that promotes the occurrence and progression of ESCA.

### FGL2 is crucial in promoting the initiation and progression of ESCA

The upregulation of FGL2 has been reported in different cancers [22]. Thus, we evaluated *FGL2* expression in esophageal cancer tissues from TCGA and esophageal cancer cell lines from the Cancer Cell Line Encyclopedia database. As shown in Supplementary Figure 1, *FGL2* expression level in esophageal cancer tissues was higher than normal tissues. However, *FGL2* expression was low in esophageal cancer cell lines. We speculate that *FGL2* is highly expressed in the tumor stroma, and that M2 macrophages in the tumor stroma secrete FGL2 to alter the TME.

**Figure 6:**
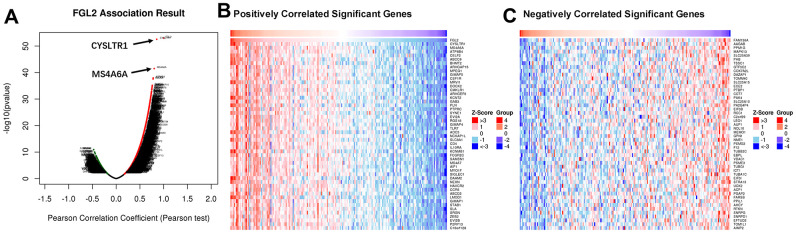
**Correlations between the levels of *FGL2* and other genes in ESCA using LinkedOmics.** (**A**) Pearson’s test was used to analyze the correlations between the levels of *FGL2* and other genes in ESCA tissues. (**B**, **C**) Heatmaps showing the top 50 genes that correlated positively and negatively with *FGL2* in ESCA. Positively correlated genes are indicated in red, while negatively correlated genes are shown in blue.

To assess the association between the levels of *FGL2* and other genes in ESCA tissues, we analyzed mRNA sequencing data from 184 ESCA patients using the function module of LinkedOmics. As shown in the volcano plot in Figure 6A, 4,078 genes exhibited significant positive correlations with *FGL2*, whereas 1,177 genes exhibited significant negative correlations with *FGL2* (false discovery rate [FDR] < 0.01). The 50 most significant gene sets that correlated positively and negatively with *FGL2* are shown in the heatmaps in Figures 6B, 6C. These results revealed the widespread impact of FGL2 on the transcriptome.

**Figure 7:**
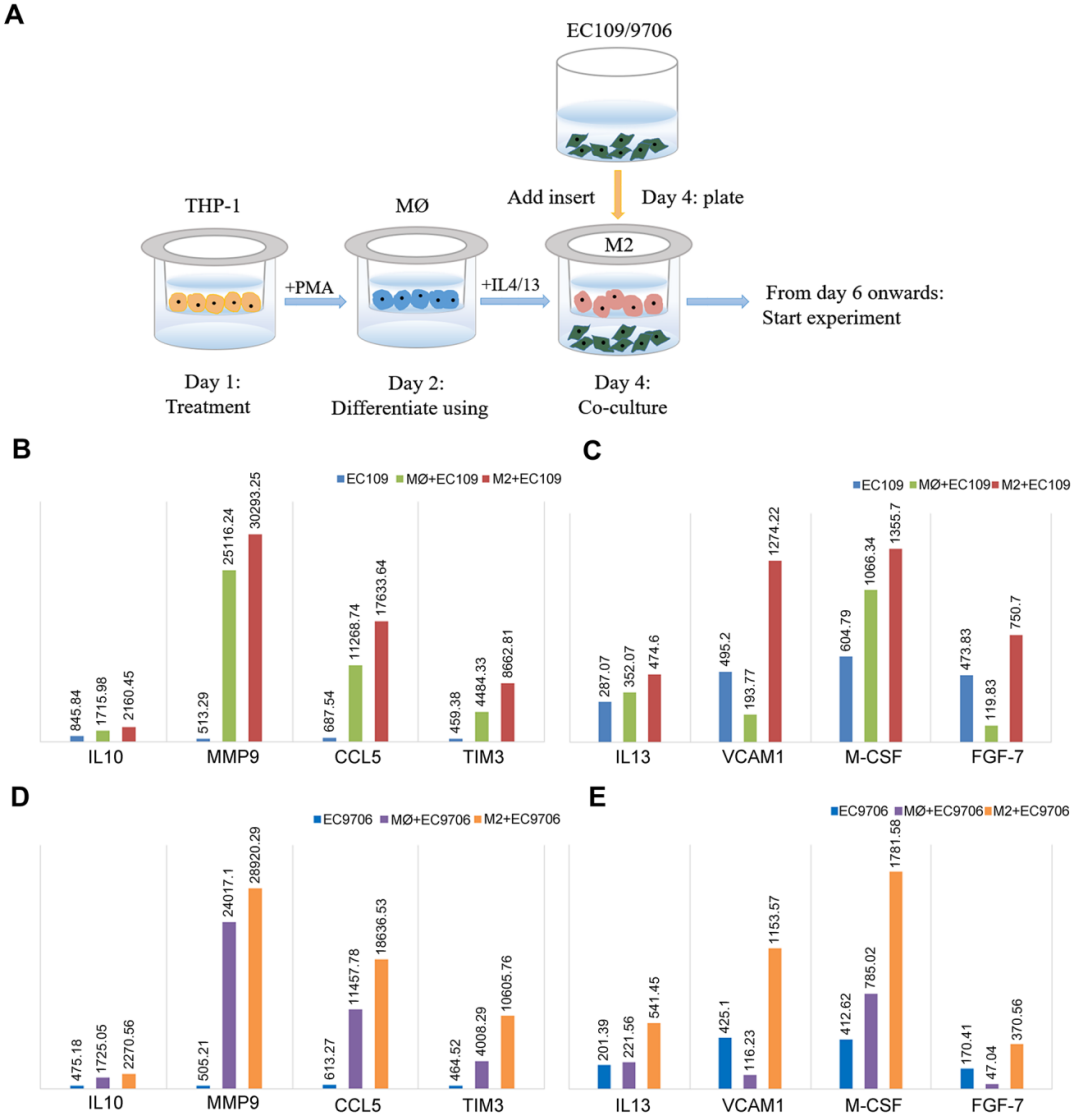
**Identification of cytokines with significantly different levels in ESCC cells co-cultured with macrophages.** (**A**) Schematic of the process of co-culturing macrophages and ESCC cells (EC109 and EC9706). (**B**, **C**) The levels of IL-10, MMP9, CCL5, TIM-3, IL-13, VCAM1, M-CSF and FGF-7 in EC109 cells cultured alone, co-cultured with M0 macrophages or co-cultured with M2 macrophages. (**D**, **E**) The levels of IL-10, MMP9, CCL5, TIM-3, IL-13, VCAM1, M-CSF and FGF-7 in EC9706 cells cultured alone, co-cultured with M0 macrophages or co-cultured with M2 macrophages. MØ, M0 macrophages; M2, M2-like TAMs.

To better understand whether FGL2 is a crucial determinant of the TME and tumor metastasis in esophageal cancer, we compared the cytokine levels among EC109/9706 (ESCC) cells cultured alone, co-cultured with M0 macrophages or co-cultured with M2 macrophages (Figure 7A). We considered a 20% change in cytokine expression to be statistically significant. The levels of interleukin (IL)-10, matrix metalloproteinase 9 (MMP9), C-C motif chemokine ligand 5 (CCL5), TIM-3, IL-13, vascular cell adhesion molecule 1 (VCAM1), macrophage colony-stimulating factor (M-CSF) and fibroblast growth factor 7 (FGF-7) were significantly greater in EC109/9706 cells co-cultured with M2 macrophages than in EC109/9706 cells cultured alone or co-cultured with M0 macrophages (Figure 7B–7E). These results indicated that M2 macrophages promote the secretion of these cytokines in the TME, thereby inducing the occurrence and progression of ESCA.

**Figure 8:**
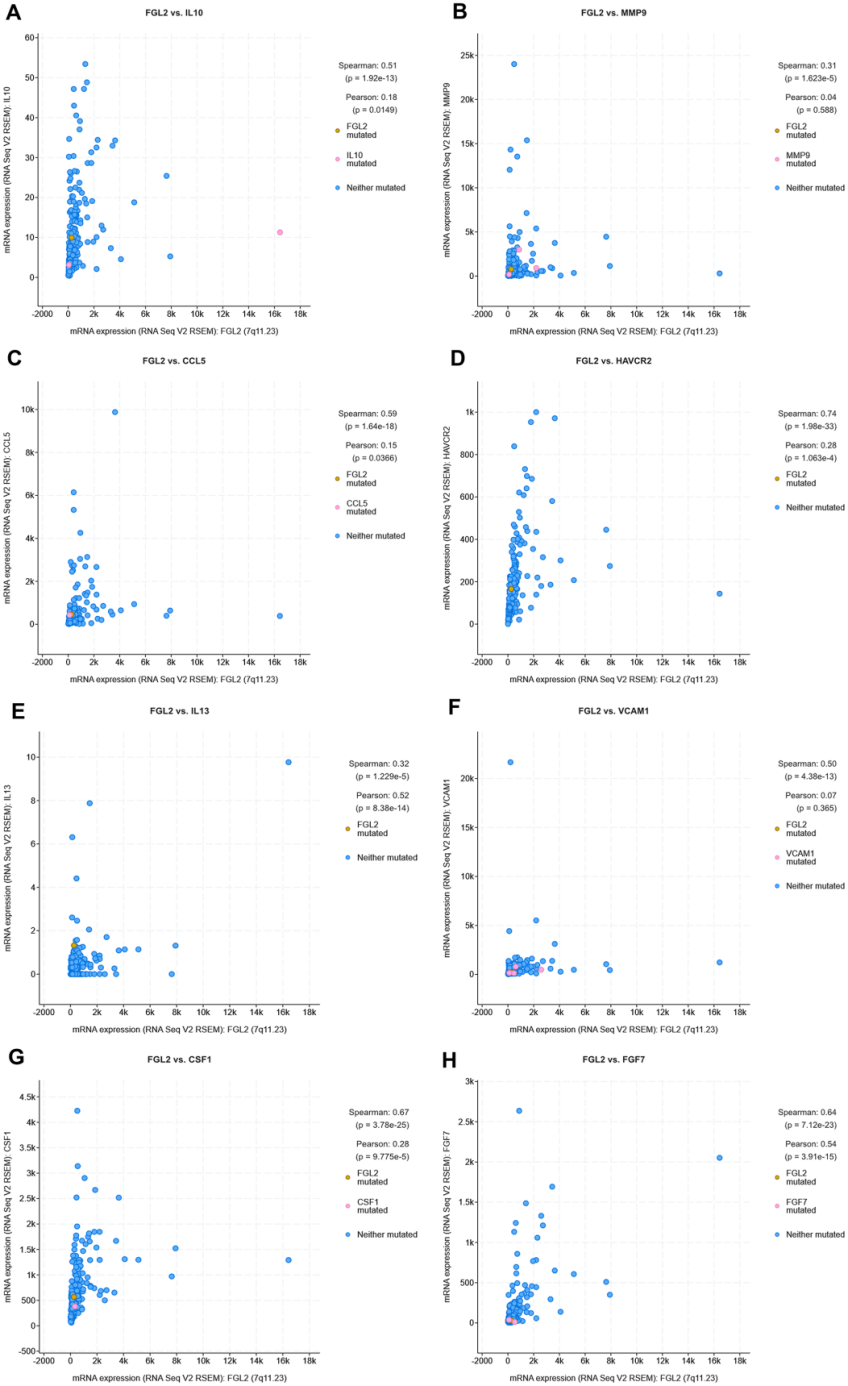
**Correlation analysis between cytokine and *FGL2* levels in ESCA tissues using cBioPortal.** (**A**–**H**) Scatterplots depicting the correlations between the levels of *FGL2* and *IL-10* (**A**), *MMP9* (**B**), *CCL5* (**C**), *TIM-3* (**D**), *IL-13* (**E**), *VCAM1* (**F**), *M-CSF* (*CSF1*) (**G**) and *FGF-7* (**H**). A Spearman’s P < 0.05 was considered statistically significant.

Next, we used cBioPortal to evaluate the correlations between the levels of *FGL2* and the cancer-promoting cytokines measured above (*IL-10*, *MMP9*, *CCL5*, *TIM-3*, *IL-13*, *VCAM1*, *M-CSF* and *FGF-7*) in ESCA tissues. As shown in the scatterplots in Figure 8, *FGL2* expression exhibited a strong positive correlation with the expression of each of these cytokines. The positive correlation between *FGL2* and *TIM-3* (*HAVCR2*), a crucial promoter of T cell exhaustion [23], suggested that high *FGL2* expression accompanies TIM-3-induced T cell exhaustion. We also assessed the correlation of *FGL2* expression with *PD-1*, *CTLA4*, *FOXP3* and *CCR8* expression in ESCA tissues in cBioPortal. *FGL2* levels correlated strongly and positively with the levels of *PD-1* and *CTLA4*, which are induced during T cell exhaustion (Supplementary Figure 2), consistent with the results in Table 2.

These results confirmed that increased *FGL2* expression in M2 macrophages alters the TME in ESCA. The correlation between the expression of *FGL2* and the presence of specific infiltrating immune cells in ESCA suggested that FGL2 is a vital contributor to immune escape and immunosuppression in the esophageal cancer microenvironment.

### Reversing TAM polarization may provide new targets for tumor immunotherapy

**Figure 9:**
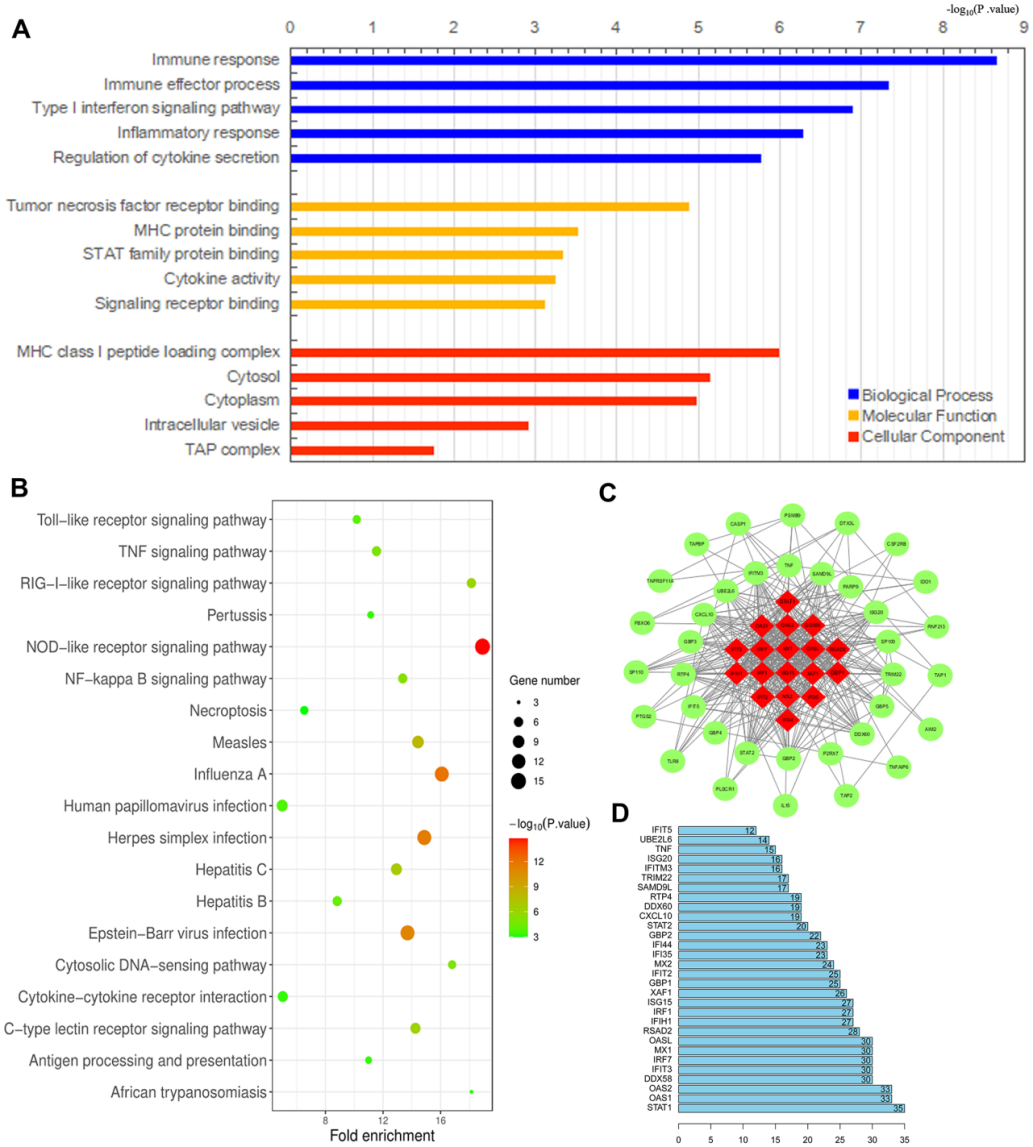
**Analysis of DEGs between M1 and M2 macrophages.** (**A**, **B**) Histogram of enriched GO terms (BP: Biological Process, MF: Molecular Function, CC: Cellular Component) (**A**) and bubble plot of enriched KEGG pathways (**B**). The rank criterion was an FDR < 0.05. The abscissa represents the percentage of DEGs enriched in each term or pathway; the size represents the number of DEGs enriched in each item or pathway; and the color represents the adjusted P-value. (**C**) PPI network constructed from 54 hub genes, with the red region representing the most closely connected module. A comprehensive Gt score > 0.7 was considered statistically significant. (**D**) Top 30 genes with the greatest degree of differential expression. The ordinate lists the gene name and the abscissa represents the degree of differential expression.

We previously demonstrated that TAMs directly promote the survival, invasion and metastasis of esophageal cancer cells [24]. To identify an effective immunotherapeutic target for ESCA, we used the WEB- based GEne SeT AnaLysis Toolkit (WebGestalt) to conduct an enrichment analysis of the GO biological functions (Biological Process, Molecular Function and Cellular Component) and KEGG pathways of the 91 DEGs identified between M1 and M2 macrophages above. The GO analysis indicated that the DEGs were primarily enriched in the Immune response Type I interferon signaling pathway, Immune effector process, Tumor necrosis factor receptor binding, Major histocompatibility complex (MHC) protein binding, STAT family protein binding, MHC class I peptide loading complex, Cytosol, Cytoplasm, etc. (Figure 9A). Furthermore, the KEGG pathway enrichment analysis demonstrated that these DEGs were enriched in the Toll-like receptor (TLR) signaling pathway, Tumor necrosis factor signaling pathway, RIG-l-like receptor signaling pathway, Pertussis, NOD-like receptor signaling pathway, Nuclear factor κB signaling pathway, Necroptosis, Measles, Influenza A, Human papillomavirus infection, etc. (Figure 9B). These results suggested potential treatment targets involved in TAM polarization that could be used to improve anti-tumor immunity.

We also performed a STRING database screening to analyze the functional interactions between the proteins encoded by the 91 DEGs. We used Cytoscape software to visualize closely connected regions in the protein-protein interaction (PPI) network, and used the MCODE plugin to identify the top hub genes constituting the most closely connected module. The PPI network included 54 nodes and 422 edges (Figure 9C). The top 30 genes among the 54 hub genes are shown in Figure 9D. These key genes could be considered during the development of novel molecular drugs for TAM-related immunotherapies to treat ESCA.

**Figure 10:**
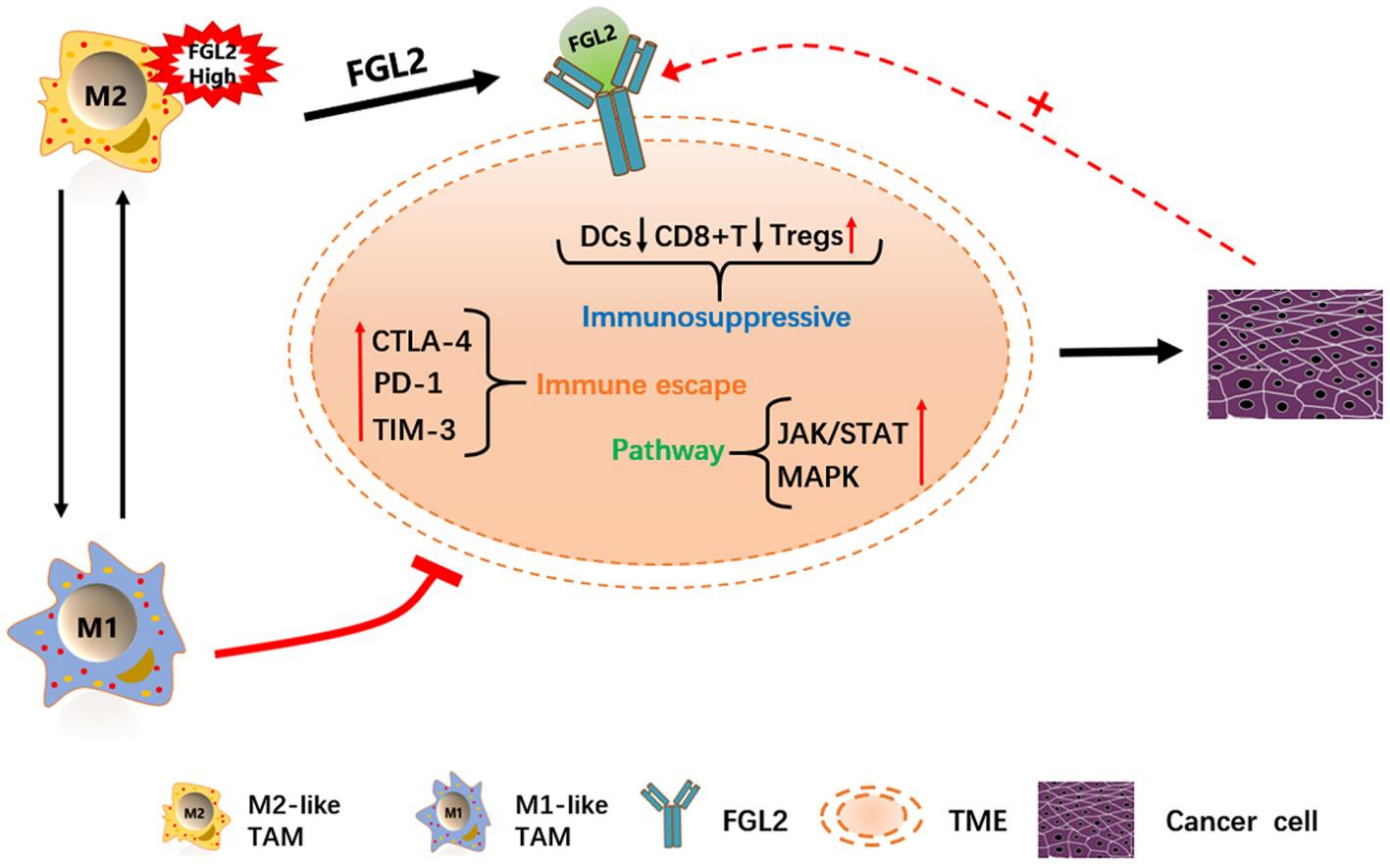
**Schematic diagram depicting the potential function of FGL2 in ESCA.** M2-like TAMs recruit infiltrating immune cells by upregulating *FGL2*, producing a microenvironment that promotes the occurrence and development of ESCA. *FGL2* levels correlate with infiltrating immune cell levels, and FGL2 promotes immune escape in the esophageal cancer microenvironment. This study has provided new targets for the development of tumor immunotherapies that treat ESCA by reversing the polarization of TAMs into M1-like TAMs.

FGL2 is already considered a potential molecular target for glioblastoma treatment [25]. Therefore, we performed a gene set enrichment analysis (GSEA) to identify functions or pathways that varied between ESCA tissues with low and high *FGL2* levels. We found that the mitogen-activated protein kinase (MAPK), Janus kinase (JAK)/STAT, TLR and other immune-related pathways were enriched in ESCA tissues with high *FGL2* levels (Supplementary Figure 3). Our results indicated that inhibiting FGL2 could potentially weaken the immunosuppressive activity of ESCA tumors to prevent their progression. A schematic diagram depicting the potential function of FGL2 in ESCA is shown in Figure 10.

## DISCUSSION

Macrophages constitute a key immune population in the TME that can prevent or promote tumor growth. M2-like TAMs have been shown to suppress anti-tumor immunity, induce angiogenesis and promote cell migration [26]. An increased number of M2-like TAMs has been observed during tumor progression and has been associated with a poor patient prognosis [27]. Our previous findings in esophageal cancer supported this notion. TAMs are an essential component of the esophageal cancer microenvironment, and an increased proportion of TAMs in ESCA has been associated with high invasiveness and a poor patient prognosis.

Increased FGL2 expression has been identified in several human tumors, including glioma [28], lymphoma [29], lung cancer [30] and liver cancer [31]. To our knowledge, the present study is the first to demonstrate the association between high *FGL2* expression in M2 macrophages and a poor prognosis (OS and RFS) in esophageal cancer patients. We found that the expression of genetic markers of M1 macrophages (*IRF5*, *INOS* and *COX2*) did not correlate with *FGL2* expression in ESCA tissues, while the expression of genetic markers of M2 macrophages (*CD163*, *VSIG4* and *MS4A6A*) correlated closely with *FGL2* expression. These results strongly suggested that M2 macrophages promote the initiation and progression of ESCA by secreting FGL2 to create an immunosuppressive TME. Moreover, our data revealed that FGL2 may direct the polarization of TAMs toward the M2 phenotype.

We also found that *FGL2* expression correlated significantly with the levels of genomic markers of exhausted T cells, Tregs, DCs, CD8+ T cells and numerous markers of T helper cells (Th1, Th2, Tfh and Th17) in ESCA tissues. A previous study demonstrated that exposure of immature DCs to soluble FGL2 inhibited the expression of MHC II molecules and CD80, thereby suppressing DC maturation and antigen presentation [32]. The binding of FGL2 to Fcγ receptor IIB was reported to hinder DC and B cell activity [17]. These may be the mechanisms whereby FGL2 alters infiltrating immune cell functions in ESCA.

Our analysis of co-expressed genes illustrated that cysteinyl leukotriene receptor 1 (*CYSLTR1*, the receptor for leukotriene D4) and *MS4A6A* levels correlated positively with *FGL2* levels in ESCA tissues. A previous study indicated that MMP9 activity and expression were reduced by a CYSLTR1 antagonist (montelukast), and that M2 macrophages enriched in leukotriene D4 promoted the migration and invasion of colon cancer cells by inducing MMP9 expression [33]. CD163, MS4A4A and MS4A6A are surface markers for M2 macrophages, and higher MS4A4A and MS4A6A levels were associated with a poorer prognosis in ovarian cancer patients [34]. Our results were in accordance with these findings.

We also found that *FGL2* expression correlated positively with IL-10, MMP9, CCL5, TIM-3, IL-13, VCAM1, M-CSF and FGF-7 expression in a co-culture of M2 macrophages with EC109/9706 cells. Our previous research indicated that MMP9 promotes the invasion and migration of ESCA cells [35]. Cytokines such as M-CSF, IL-10, IL-13 and IL-4 induce the polarization of TAMs into M2 macrophages, which produce anti-inflammatory cytokines such as transforming growth factor β and IL-10 [36]. CCL5, M-CSF and CCL2 are involved in monocyte recruitment, and M-CSF is also involved in macrophage survival [37]. CCL5 and VCAM1 have been shown to promote tumor progression and metastasis in ESCC [38, 39]. Moreover, FGF-7 is known to induce cell migration and invasion by activating nuclear factor αB [40]. Thus, our results suggested that M2 macrophages stimulate the expression of cancer-promoting factors in the TME by secreting FGL2, that elevated *FGL2* expression drives the polarization of TAMs toward the M2 phenotype, and that FGL2 promotes the recruitment of particular infiltrating immune cells in ESCA.

FGL2 is mainly expressed by activated endothelial cells, macrophages, T cells and tumor cells. FGL2 derived from the tumor matrix was reported to promote the occurrence and development of lung cancer [30]. Interestingly, FGL2 is primarily but not exclusively expressed in the immune cells, tumor cells and tumor stroma of the TME, and can also be produced by hematopoietic cells [41]. Notably, we found that *FGL2* expression correlated more strongly with TAM and M2 macrophage levels in normal tissues than in ESCA tumor tissues (Table 3). Nevertheless, our results suggested that M2 macrophages in the TME secrete FGL2 to exert immunosuppressive effects. Infiltrating immune cells and tumor cells may also secrete FGL2 to promote the polarization of TAMs into M2 macrophages, forming an important carcinogenic loop that promotes the initiation and progression of ESCA. Thus, inhibiting FGL2 may reduce the polarization of M2 macrophages, weaken immunosuppression and prevent the progression of ESCA. Further investigation is required to assess the potential of FGL2 as a therapeutic target and immunomodulator in ESCA, and to determine whether FGL2 levels in peripheral blood can be used as a tumor marker for the early screening and diagnosis of this disease.

Cancer immunotherapies are typically designed to reduce immunosuppression or to restore and enhance anti-tumor immunity. Broadly, anti-cancer treatment efficacy can be enhanced through the inhibition of TAM recruitment to the tumor site or through the reprogramming of TAMs into anti-tumor M1 macrophages. TAMs express the ligands for checkpoint molecules in T cells, including PD-L1, PD-L2, VISTA and B7H4, which are upregulated in response to stimuli such as hypoxia and cytokines [42, 43]. In a pancreatic cancer preclinical model, Ibrutinib (an inhibitor of Bruton’s tyrosine kinase) was used to reset M0 macrophages toward an M1-like phenotype that promoted CD8+ T cell cytotoxicity, and this approach is currently being evaluated in combination with checkpoint inhibitors [44]. In addition, several preclinical studies have demonstrated that agonists of the TLRs (TLR3, TLR7/8 and TLR9) in macrophages may enhance anti-tumor treatment efficacy by polarizing M0 macrophages into Th1 and M1-like TAMs and inhibiting tumor progression [45]. In the present study, we found that *TNFRSF11A*, *HGSNAT*, *MCM6*, *RNASE6*, *FAM101B*, *ACPP*, *EMB* and *NCAPH* were significantly upregulated in M2 macrophages compared with M1 macrophages. The most highly upregulated gene was *TNFRSF11A*, which has been reported to promote cervical cancer cell migration, invasiveness and proliferation [46]. Thus, inhibiting these genes may reduce the polarization of TAMs into M2 macrophages and weaken their immunosuppressive effects.

In addition, our GO analysis revealed that the significantly DEGs between M1 and M2 macrophages were primarily enriched in the type I interferon signaling pathway. When the type I interferon receptor (interferon-a/β receptor, IFNAR) binds to its ligand, activated IFNAR1 binds to tyrosine kinase 2, inducing IFNAR2 to bind to JAK1, ultimately inducing the JAK/STAT, MAPK and phosphoinositide 3-kinase/mammalian target of rapamycin signaling pathways [47]. The activation of these pathways is associated with the occurrence, growth, apoptosis inhibition and metastasis of tumor cells. In our PPI analysis, the majority of the genes in the most closely connected module were significantly upregulated in M1 macrophages, including *STAT1*, *OAS1*, *OAS2* and *DDX5*; thus, developing small molecule agonists for these proteins may enhance anti-tumor immunity.

In conclusion, our results suggested that *FGL2*, which is highly expressed by M2 macrophages, could be considered as a tumor marker for early ESCA diagnosis and an effective immunotherapeutic target for ESCA treatment. The DEGs we identified between M1 and M2 macrophages could also be used to reprogram TAMs into anti-tumor M1 macrophages for cancer immunomodulation therapy. However, due to the limitations of our analysis, subsequent experimental verification is required.

## MATERIALS AND METHODS

### Evaluation of TIICs in ESCA

In this study, we used expression data from TCGA ESCA cohorts. The CIBERSORT analytical tool contains 547 genes that distinguish 22 human hematopoietic cell phenotypes, including seven T cell types, naive and memory B cells, macrophages of different phenotypes, plasma cells, natural killer cells and myeloid subsets [48]. We used this tool to estimate the proportions of the 22 TIICs in both ESCA samples and normal samples. To assess the accuracy of the deconvolution results, CIBERSORT derives a P-value for each sample. A CIBERSORT P-value < 0.05 was considered statistically significant and was used as the sample inclusion criterion. In total, 78 samples (2 normal samples and 76 ESCA samples in TCGA) were included. The CIBERSORT software package was obtained from the developers and combined with R (R.3.6.1). We used the default signature matrix with ESCA permutations for analysis.

### Microarray data

Genes were screened using the following datasets from the GEO database (http://www.ncbi.nlm.nih.gov/geo): the GSE57614 series [49] and the GSE36537 series [50] on the GPL6480 platform (Agilent-014850 Whole Human Genome Microarray 4x44K G4112F), the GSE5099 series [51, 52] on the GPL96 platform (Affymetrix Human Genome U133A Array) and the GPL97 platform (Affymetrix Human Genome U133B Array), and the GSE95405 series on the GPL570 platform (Affymetrix Human Genome U133 Plus 2.0 Array). According to the annotation information in each platform, probes were converted into corresponding gene symbols. The GSE57614 dataset contained nine samples each of M0 macrophages, M1 macrophages and M2 macrophages. GSE36537 contained nine samples each of M0 macrophages, M1 macrophages and M2 macrophages. GSE5099 contained six samples each of M0 macrophages, M1 macrophages and M2 macrophages. GSE95405 contained three samples each of M1 macrophages and M2 macrophages.

### Identification of DEGs

GEO2R is an interactive web tool that allows users to compare two or more groups of samples in a GEO series in order to identify genes that are differentially expressed across experimental conditions. We used GEO2R (https://www.ncbi.nlm.nih.gov/geo/geo2r/) to identify DEGs between M0 macrophages and M2 macrophages samples, and between M1 and M2 macrophages samples. The parameters used for the comparison of M0 macrophages and M2 macrophages samples were ‘‘adjusted P-value < 0.05, |logFC| ≥ 1.0’’. The parameters used for the comparison of M1 and M2 macrophages samples were ‘‘adjusted P-value < 0.01, |logFC| ≥ 1.0’’. The Venn online tool (http://bioinformatics.psb.ugent.be/webtools/Venn/) was used to identify significantly DEGs, and overlapping DEGs in the Venn diagram were retained for further analysis.

### Selection and analysis of hub genes in macrophage and M2 macrophage samples

The cBioPortal (https://www.cbioportal.org/) is a web resource for exploring, analyzing and visualizing multidimensional cancer genomics data [53]. We used cBioPortal to analyze the expression of hub genes in ESCA samples.

Kaplan-Meier Plotter (http://kmplot.com/analysis/) can be used to assess the effect of any gene or gene combination on survival in breast cancer, lung cancer, ovarian cancer, gastric cancer, colon cancer, prostate cancer, melanoma and 14 other tumor types. The database includes over 50,000 samples that were assessed using gene arrays and RNA sequencing [54]. We used Kaplan-Meier Plotter to determine the correlation of hub gene expression (*FGL2*, *ERI1* and *WNT5B*) with the OS and RFS of ESCC patients, together with the HR and log-rank P-value.

The TIMER database is a comprehensive resource for the systematic analysis of immune infiltrates across diverse cancer types (https://cistrome.shinyapps.io/timer/) [55], and can be used to infer the abundance of TIICs from gene expression profiles according to a previously published deconvolution method [56]. Using the gene modules in TIMER, we assessed the correlation of hub gene expression with the abundance of immune infiltrates such as CD4+ T cells, CD8+ T cells, macrophages, B cells, DCs and neutrophils. We also analyzed the association of hub gene expression with ESCA purity [57].

### Correlation analysis between the levels of *FGL2* and TIICs

We used the correlation modules in TIMER to explore the correlations between the levels of *FGL2* and genetic markers of TIICs (monocytes, TAMs, M1 macrophages, M2 macrophages, T cells [general], CD8+ T cells, B cells, Tregs, Th1 cells, Th2 cells, exhausted T cells, etc.). These genetic markers have been referenced in previous studies [58–60]. The correlation module generates an expression scatterplot between a pair of user-defined genes, as well as the Spearman’s correlation coefficient and statistical significance. Gene levels were calculated as log2 RSEM data.

The online database GEPIA (http://gepia.cancer-pku.cn/index.html) [61], a web server for cancer and normal gene expression profiling and interactive analyses, was used to confirm the correlations between the levels of *FGL2* and markers of macrophages of different phenotypes. ESCA and normal tissue datasets were analyzed, and Spearman’s correlation coefficients were determined.

### Correlation analysis between the levels of *FGL2* and co-expressed genes and cytokines, and GSEA based on *FGL2* expression

The publicly available LinkedOmics database (http://www.linkedomics.org/login.php) includes multi-omics data from all 32 TCGA cancer types, as well as mass spectrometry-based proteomics data generated by the Clinical Proteomics Tumor Analysis Consortium for TCGA breast, colorectal and ovarian tumors [62]. We used this database to identify DEGs in correlation with *FGL2* in the ESCA cohort from TCGA (n=184). The Pearson method was used to determine the correlation coefficients. The LinkFinder also generates statistical expression plots for individual genes, including volcano plots and heatmaps. The correlations between the levels of *FGL2* and cytokines in samples from TCGA were analyzed using cBioPortal. The Cancer Cell Line Encyclopedia (CCLE) have provided a rigorous framework with which to study genetic variants (https://portals.broadinstitute.org/ccle) [63]. *FGL2* mRNA levels in esophageal cancer cell lines were determined using the CCLE.

GSEA can be used to determine whether a predefined gene set exhibits consistently significant differences in two biological states [64]. We used GSEA software (version: 4.0.0) to identify functions or pathways that differed between ESCA samples with low and high *FGL2* mRNA levels. Differences were screened based on a normalized P-value < 0.05 and an FDR < 0.05, and 1000 gene set permutations were used in each analysis.

### Macrophage and ESCC cell co-culture

Human ESCC (EC109/9706) cells and leukemia THP-1 monocytes were obtained from Fuxiang Biological Company (Shanghai, China). All cells were maintained in Roswell Park Memorial Institute 1640 medium (Gibco, Carlsbad, CA, USA) supplemented with 10% fetal bovine serum (Gibco), and were cultured in a humidified incubator containing 5% CO_2_ at 37°C. Phorbol-12-myristate-13-acetate (Beijing 4A Biotech Co., Ltd) was added to the THP-1 monocytes to a final concentration of 40 ng/mL. Approximately 36 h later, the cell morphology was observed under an inverted microscope to confirm that the THP-1 monocytes had been induced to differentiate into M0 macrophages. Then, IL-4 and IL-13 (PeproTech, USA) were added to the M0 macrophages to final concentrations of 8 ng/mL and 4 ng/mL, respectively. After 48 h, the cell morphology was observed under an inverted microscope to confirm that the macrophages had been induced to differentiate into M2 macrophages. The EC109/9706 cells and M2 macrophages were respectively cultured in the upper and lower chambers of six-well plates (Corning Inc., Corning, NY, USA), each with 2 mL of culture medium. The cells were placed in an incubator for 48 h to establish a non-contact co-culture. The non-contact co-culture of EC109/9706 cells and M0 macrophages was established in the same way.

### Human cytokine antibody array

We used the Human XL Cytokine Array Kit (R & D; Cat ARY022B) to detect the expression of various cytokines in cell co-culture samples. For each Array unit, 200-500 μg of cell culture supernatant was used. The protein chip was blocked with 2 mL of Array Buffer and incubated for 1 h on a shaker. The samples were diluted with Array Buffer to a volume of 1.5 mL per membrane and then incubated with the Array protein membrane at 4 °C overnight. The membrane was washed three times with wash buffer, treated with a diluted detection antibody and incubated at room temperature for 1 h. The membrane was then washed, treated with streptavidin-horseradish peroxidase, incubated for 30 min and shaken at room temperature. After the membrane was washed, 1 mL of the chemical color reagent was added to each well, and the color was developed on a chemiluminescence imager. Professional software was used to process and analyze the data.

### Analysis of DEGs between M1 and M2 macrophages samples

WebGestalt (http://www.webgestalt.org) [65], one of the most widely used GSEA tools, was used to analyze the KEGG pathways and GO terms (Biological Process, Molecular Function and Cellular Component) of the DEGs between M1 and M2 macrophages. The rank criterion was an FDR < 0.05. The most important module in the PPI network was built using the STRING online database (http://string-db.org, Version: 11.0) [66]. A comprehensive Gt score > 0.7 was considered statistically significant. Cytoscape [67] was used to cluster the resulting network to reveal closely connected regions.

### Data availability

All datasets generated for this study are included in the manuscript.

### Ethics statement

As the data (TCGA and GEO datasets, etc.) are publicly available, no ethical approval was required.

## Supplementary Material

Supplementary Figures
