# Supplementary material for: Tumor-associated macrophage polarization promotes the progression of esophageal carcinoma
Source: Aging (Albany NY). 2020 Dec 15;13(2):2049–72. doi: 10.18632/aging.202201 (PMC7880404; doi:10.18632/aging.202201)
Supplement: Supplementary Figures [file aging-13-202201-s001.pdf]

SUPPLEMENTARY FIGURES

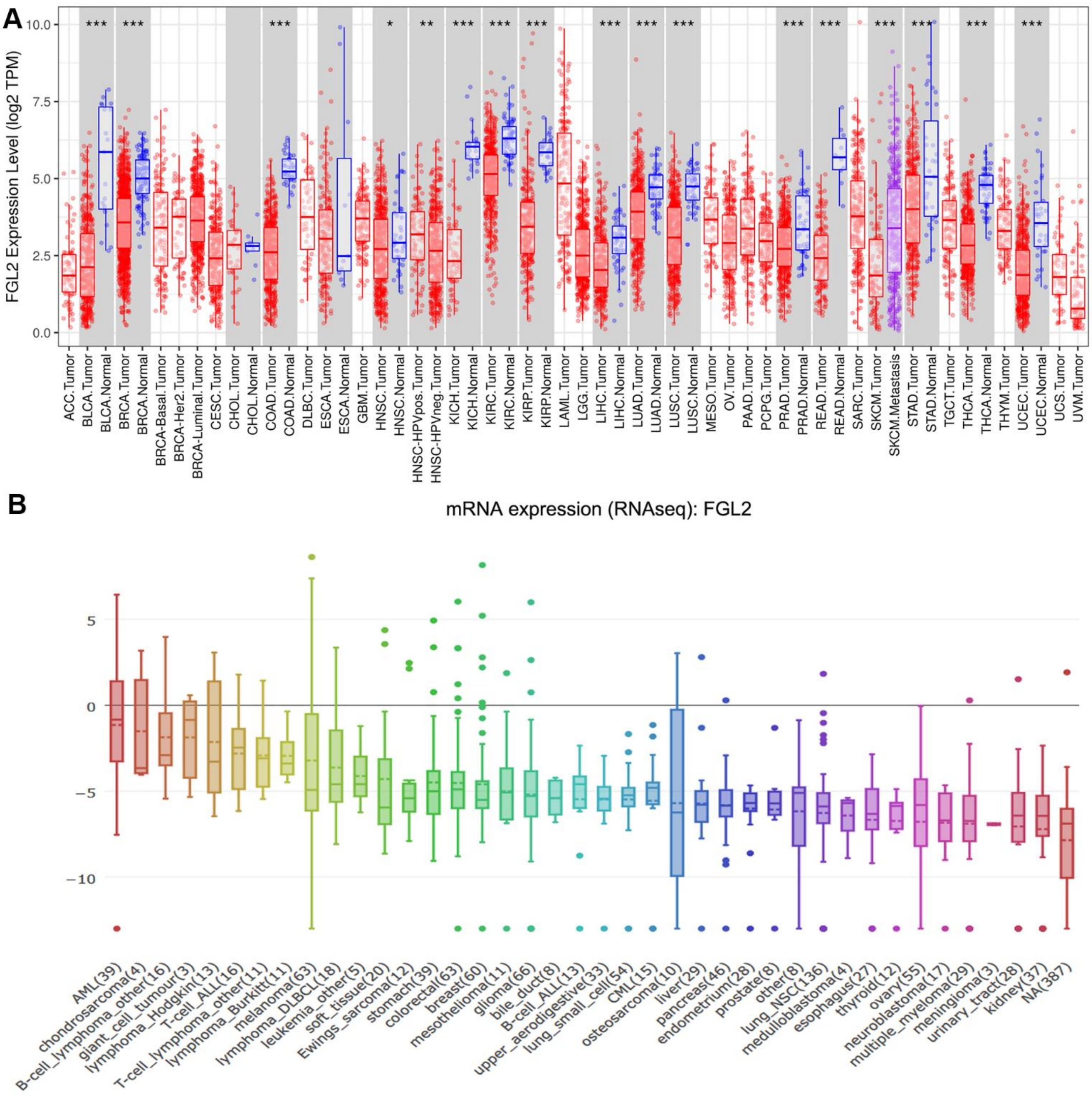

Supplementary Figure 1. *FGL2* levels in tumor tissues and cell lines. (**A**) Human *FGL2* levels in different tumor types from TCGA were determined using TIMER (*P < 0.05, **P < 0.01, ***P < 0.001). (**B**) *FGL2* mRNA levels in cancer cell lines were determined using the Cancer Cell Line Encyclopedia.

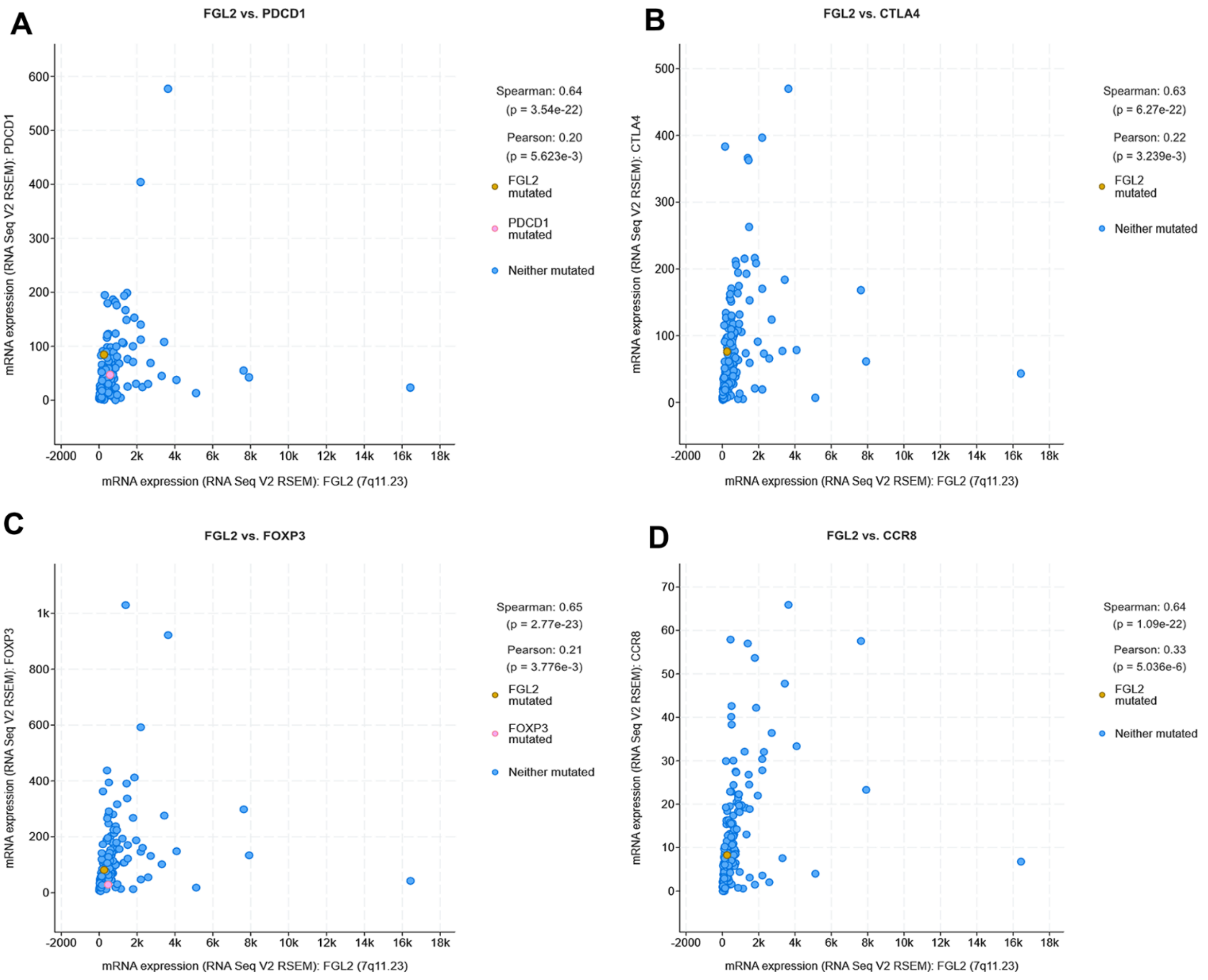

Supplementary Figure 2. Correlation analysis between the levels of *FGL2* and genetic markers in ESCA using cBioPortal. (**A**–**D**) Scatterplots depicting the correlations between the levels of *FGL2* and *PD-1* (**A**), *CTLA4* (**B**), *FOXP3* (**C**) and *CCR8* (**D**). A Spearman's $P < 0.05$ was considered statistically significant.

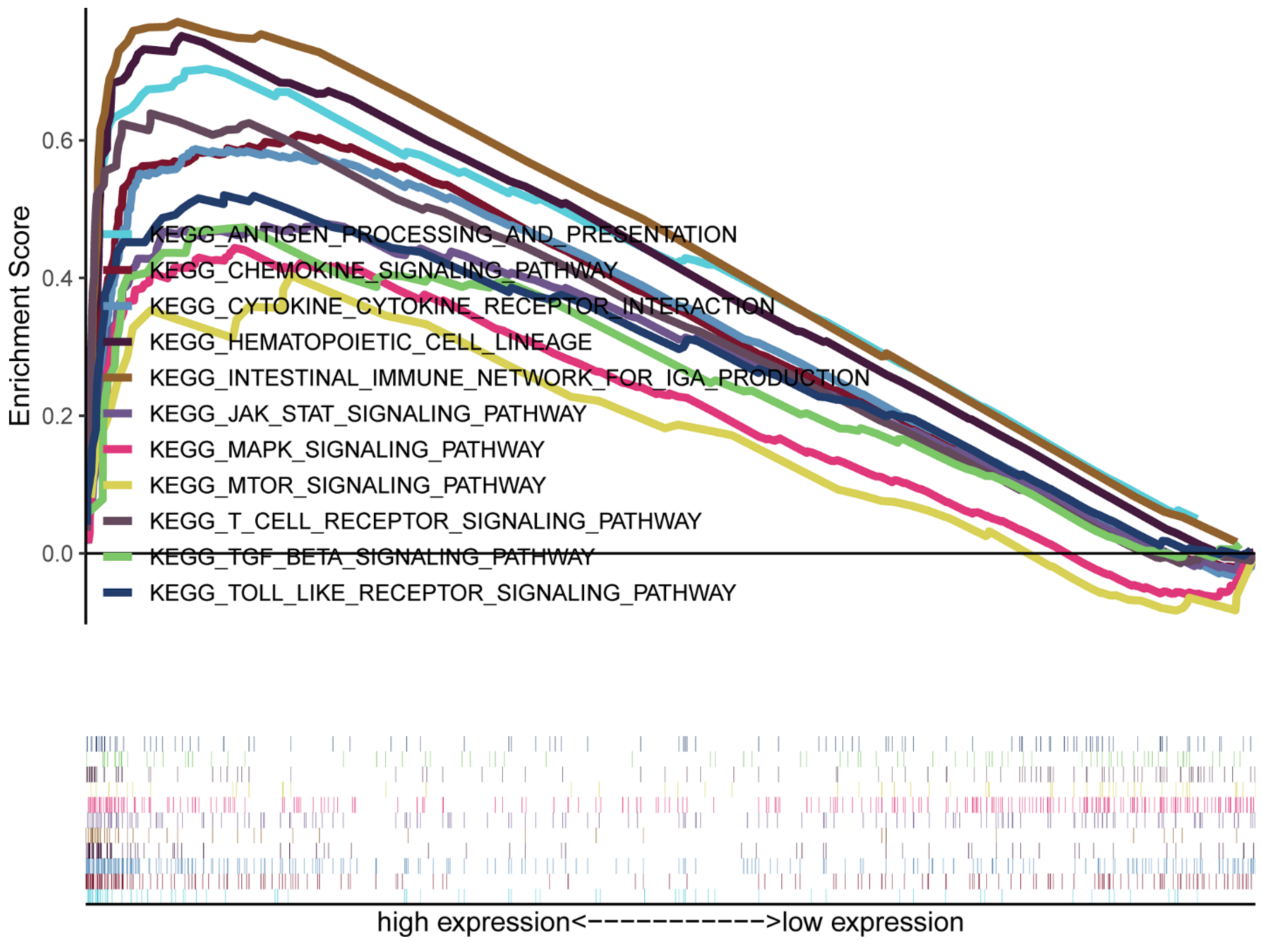

Supplementary Figure 3. GSEA results based on *FGL2* expression in ESCA samples from TCGA.
